# Age‐dependent decrease in TRPM4 channel expression but not trafficking alters urinary bladder smooth muscle contractility

**DOI:** 10.14814/phy2.14754

**Published:** 2021-02-24

**Authors:** Sarah E. Maxwell, M. Dennis Leo, John Malysz, Georgi V. Petkov

**Affiliations:** ^1^ Department of Pharmaceutical Sciences College of Pharmacy University of Tennessee Health Science Center Memphis TN USA; ^2^ Department of Pharmacology College of Medicine University of Tennessee Health Science Center Memphis TN USA; ^3^ Department of Urology College of Medicine University of Tennessee Health Science Center Memphis TN USA

**Keywords:** channel trafficking, detrusor, ion channel, maturation, Western blot

## Abstract

During development, maturation, or aging, the expression and function of urinary bladder smooth muscle (UBSM) ion channels can change, thus affecting micturition. Increasing evidence supports a novel role of transient receptor potential melastatin‐4 (TRPM4) channels in UBSM physiology. However, it remains unknown whether the functional expression of these key regulatory channels fluctuates in UBSM over different life stages. Here, we examined TRPM4 channel protein expression (Western blot) and the effects of TRPM4 channel inhibitors, 9‐phenanthrol and glibenclamide, on phasic contractions of UBSM isolated strips obtained from juvenile (UBSM‐J, 5–9 weeks old) and adult (UBSM‐A, 6–18 months old) male guinea pigs. Compared to UBSM‐J, UBSM‐A displayed a 50–70% reduction in total TRPM4 protein expression, while the surface‐to‐intracellular expression ratio (channel trafficking) remained the same in both age groups. Consistent with the reduced total TRPM4 protein expression in UBSM‐A, 9‐phenanthrol showed lower potencies and/or maximum efficacies in UBSM‐A than UBSM‐J for inhibiting amplitude and muscle force of spontaneous and 20 mM KCl‐induced phasic contractions. Compared to 9‐phenanthrol, glibenclamide also attenuated both spontaneous and KCl‐induced contractions, but with less pronounced differential effects in UBSM‐A and UBSM‐J. In both age groups, regardless of the overall reduced total TRPM4 protein expression in UBSM‐A, cell surface TRPM4 protein expression (~80%) predominated over its intracellular fraction (~20%), revealing preserved channel trafficking mechanisms toward the cell membrane. Collectively, this study reports novel findings illuminating a fundamental physiological role for TRPM4 channels in UBSM function that fluctuates with age.

## Graphical abstract

In this study, based on an experimental animal model, we revealed a novel age‐dependent role of TRPM4 channels in UBSM function. Specifically, there was higher expression of TRPM4 channel protein in UBSM from juvenile guinea pigs associated with increased inhibitory effects of a TRPM4 channel blocker on contractility compared to UBSM from older adult animals. Future comparative studies on human UBSM isolated from patients with or without OAB from various age groups will provide insights as to if TRPM4 channel dysfunction is a contributing factor to OAB that increases with age.

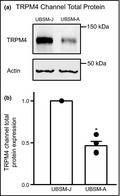

## 1 INTRODUCTION

Urinary bladder smooth muscle (UBSM) is directly regulated by myogenic and neurogenic activity. An increase in UBSM contractility is often associated with overactive bladder (OAB), a condition that affects millions of elderly patients worldwide, reducing the overall quality of life (Andersson & Schroder, 2004; Andersson & Wein, 2004). In UBSM cells, ion channels play key regulatory roles in UBSM excitation and contraction. UBSM K^+^ channel activation facilitates UBSM cell hyperpolarization and decreased UBSM cell excitability and thus tissue relaxation (Hristov et al., 2012, 2014; Malysz & Petkov, 2020; Parajuli et al., 2012; Petkov et al., 2001; Provence et al., 2015, 2018). Alternatively, the opening of voltage‐gated Ca^2+^ channels and potentially non‐selective cation channels promotes UBSM cell depolarization and increased UBSM cell excitability and therefore tissue contractility (Klockner & Isenberg, 1985; Malysz & Petkov, 2020; Sui et al., 2003, 2007; Thorneloe & Nelson, 2004; Wellner & Isenberg, 1993).

OAB becomes more prevalent with increasing age in both males and females (Andersson & Arner, 2004). On a related note, age‐dependent and maturation‐related changes in muscarinic receptor function have been observed in the UBSM of various animal species (Andersson & Schroder, 2004; Kolta et al., 1984; Ordway et al., 1986). One study based on muscarinic ligand binding experiments found higher expersion levels of muscarinic receptors in older rat urinary bladders which was linked to an increased contractile response to muscarinic agonists (Kolta et al., 1984). Another study using segments of the rat bladder body and base reported no age dependency in functional responses but reported an increase in muscarinic ligand binding in 16‐month‐old rats compared to 7‐month‐old rats. However, a decrease in muscarinic ligand binding was then reported for 27‐month‐olds compared to rats 16 months of age (Ordway et al., 1986). Both the aforementioned studies used juvenile animals not yet able to reproduce as the youngest age group, making them very relevant for age studies. A study on transient receptor potential vanilloid 4 (TRPV4) channels observed increased expression in aged male guinea pig UBSM (24–36 months) compared to younger animals (2–5 months; Roberts et al., 2019). In human UBSM isolated strips from older patients, electrical field stimulation (EFS)‐induced neuronal contractions displayed an increased atropine‐resistant (purinergic) component and preserved cholinergic component (Yoshida et al., 2001). UBSM from patients with either outflow obstructed bladders or OAB showed a reduction in functional innervation that was age‐dependent; however, UBSM contractility and excitability in response to direct EFS did not differ (Fry et al., 2011). These studies collectively demonstrate age differences in functional response and channel expression of UBSM.

Transient receptor potential melastatin 4 (TRPM4) channels have been recognized as functional regulators of UBSM excitability and contractility (Hristov et al., 2016; Kullmann et al., 2018; Parajuli et al., 2013; Smith, Hristov, et al., 2013; Smith, Parajuli, et al., 2013). Highly permeable to monovalent cations, TRPM4 channels are not permeable to anions or multivalent cations (Nilius & Owsianik, 2011; Nilius et al., 2003; Wang et al., 2018). While TRPM4 channels have been identified in UBSM of mice (Kullmann et al., 2018), rats (Parajuli et al., 2013; Smith, Parajuli, et al., 2013), guinea pigs (Smith, Hristov, et al., 2013), and human bladder (Hristov et al., 2016), their trafficking and subcellular expression profile has never been examined, and more specifically, their surface/intracellular ratio in UBSM. The commonly utilized TRPM4 channel inhibitors, 9‐phenanthrol and glibenclamide, have IC_50_ values of approximately 20–40 μM for recombinant TRPM4 channels expressed in HEK‐293 cells (Grand et al., 2008; Ozhathil et al., 2018). Unlike 9‐phenanthrol, glibenclamide can interact with the sulfonylurea receptor (SUR) subunit to inhibit SUR‐TRPM4 channel complexes (Demion et al., 2007; Ozhathil et al., 2018). Glibenclamide can also inhibit TRPM4 channels directly (Demion et al., 2007; Ozhathil et al., 2018). In rats, glibenclamide has been shown to promote urine storage increasing void volume and reducing voiding frequency using in vivo cystometry utilizing different urinary bladder disease‐induced models (Hughes, Hill, et al., 2016; Hughes, Kennis, et al., 2016; Hughes et al., 2014).

The present study aimed to determine whether TRPM4 channel expression, trafficking, and function in UBSM change during maturation. As Hartley‐Albino guinea pigs have an average lifespan of 4–5 years and males can mate as young as 3 months of age (Harkness et al., 2002), the juvenile guinea pigs at a maximum of 9 weeks of age are younger than breeding age while the adult group at a minimum of 6 months would be fully matured. For protein expression, we utilized Western blot analysis (for both total and surface/plasma membrane vs intracellular fractions) in mucosa‐free UBSM preparations from juvenile (UBSM‐J) and adult (UBSM‐A) homogenates. Such TRPM4 channel protein studies have not yet been done in UBSM of any age group. Studies of UBSM‐J and UBSM‐A contractility with TRPM4 channel inhibitors, 9‐phenanthrol and glibenclamide, provided data as to how UBSM function changes with age. We reveal novel findings of increased responses to TRPM4 channel inhibition on UBSM contractility in UBSM‐J compared to UBSM‐A that correlated with decreased total TRPM4 channel expression in UBSM‐A. Additionally, the preferential surface expression of TRPM4 channels, independent of age, reveals how mechanistically TRPM4 channels can control UBSM cell excitability and function.

## 2 MATERIALS AND METHODS

### 2.1 UBSM tissue harvesting

All experiments were conducted in accordance with the Animal Use Protocol No. 17‐075.0 which was reviewed and approved by the University of Tennessee Health Science Center (Memphis, TN). A total of 46 male Hartley‐Albino guinea pigs (Charles River, Strain Code: 051) either juvenile (5–9 weeks old; *N* = 23; median weight: 511 g, 25th percentile: 447.5 g, 75th percentile: 574 g) or adult (6–18 months old; *N* = 23; median weight: 976 g, 25th percentile: 913.5 g, 75th percentile: 1077 g) were used in this study. They were euthanized with isoflurane (Forane®, Baxter) or CO_2_ followed by thoracotomy after which the whole bladder was removed by cutting above the bladder neck and transferred to a Petri dish containing dissection solution (see *Solutions and Compounds* section for composition). The whole bladder was excised, and the mucosa including the urothelium was removed. UBSM strips (5–10 mm long and 2–4 mm wide) were cut for isometric tension recordings and Western blot protein analysis.

### 2.2 Cell surface biotinylation and Western blotting

Cellular localization of TRPM4 protein in UBSM was quantitated using a cell surface biotinylation assay. Surface biotinylation of live tissue was performed as previously described with slight modifications (Leo et al., 2015). UBSM strips were incubated in a 1 mg/ml mixture of both EZ‐Link Sulfo‐NHS‐LC‐LC‐Biotin and Maleimide‐PEG2‐Biotin reagents (Thermo Scientific Inc.) at room temperature for 1 h in whole‐cell buffer solution of the following composition (mM): 134 NaCl, 6 KCl, 2 CaCl_2_, 1 MgCl_2_, 10 HEPES, and 10 glucose (pH 7.4). Penetration of biotinylation through UBSM was confirmed via immunohistochemistry (see Figure S2, https://doi.org/10.6084/m9.figshare.13611383.v1). UBSM strips were then briefly washed with 100 mM glycine dissolved in PBS (pH 7.4) to remove any unbound biotin and lysed and homogenized in a buffer of the following composition: 50 mM Tris HCl, 150 mM NaCl, 5 mM EDTA, 1% Triton X‐100, and 0.1% SDS. After centrifugation at 9000 rpm for 5 min, the supernatant was collected and stored at −20°C for further processing.

Total protein was quantified as described previously (Henkel & Bieger, 1994). Fifty micrograms of total protein from each sample was used for Avidin (Monomeric Avidin, Pierce) pull‐down of biotinylated surface proteins. Following centrifugation at 12,000 rpm for 2 min, the supernatant was collected as the non‐biotinylated (intracellular) protein fraction. Biotinylated proteins bound to the Avidin beads were eluted by boiling the beads in 2x Laemmli buffer containing 2% 2‐mercaptoethanol. Surface and intracellular protein fractions of each sample were run in contiguous lanes on a 7.5% SDS‐PAGE gel and were transferred onto nitrocellulose membranes. After blocking the membranes with 5% non‐fat milk, blots were probed with rabbit polyclonal anti‐TRPM4 antibody (1:500, Thermo Scientific Inc., RRID : A B_2689247). Surface and intracellular TRPM4 channel bands were analyzed using Quantity One (Bio‐Rad) and are expressed as a percent of total protein.

Western blotting for total protein was done following standard protocols. Fifty micrograms of total protein was boiled for 3 min with 4x Laemmli buffer containing 2% 2‐mercaptoethanol and separated on 7.5% SDS‐PAGE gels, transferred onto nitrocellulose membranes, and probed with rabbit polyclonal anti‐TRPM4 antibody (1:500, AVIVA Systems Biology, RRID : A B_2048588) (Kuras et al., 2012). We have confirmed the specificity of the TRPM4 antibody for Western blot using TRPM4 antigenic peptide (see Figure S1, https://doi.org/10.6084/m9.figshare.13611383.v1). The secondary antibody was goat anti‐rabbit HRP conjugate (Thermo Scientific Inc., RRID : A B_228333). Bound antibody was detected using ECL chemiluminescent substrate (Thermo Scientific Inc.) and a ChemiDoc system (Bio‐Rad). Images were analyzed using Quantity One software (Bio‐Rad).

### 2.3 Isometric UBSM tension recordings

Isometric tension recordings of UBSM isolated strips were performed as previously described (Hristov et al., 2016; Malysz et al., 2014; Smith, Hristov, et al., 2013; Smith, Parajuli, et al., 2013). UBSM strips were mounted between a stationary hook and a force‐displacement transducer and placed in tissue baths filled with Ca^2+^‐containing physiological salt solution (PSS) thermostatically controlled to 37°C. UBSM strips were mounted and stretched to ∼10 mN (1 g) initially and then rinsed with fresh PSS every 15 min for 45–60 min. Tetrodotoxin (1 µM), a neuronal Na^+^ channel blocker, was added to the baths to block any neuronal activity. Following the development of stable spontaneous phasic contractions (after at least 1 h equilibration with tetrodotoxin present), increasing concentrations of 9‐phenanthrol or glibenclamide were applied cumulatively at 10‐ to 12‐min intervals. For 20 mM KCl‐induced phasic contractions, 15.3 mM KCl was added to the extracellular PSS, and contractions were allowed to stabilize in the presence of tetrodotoxin for at least an hour.

### 2.4 Solutions and Compounds

The dissection solution with nominal Ca^2+^ had the following composition (mM): 80 monosodium glutamate, 55 NaCl, 6 KCl, 10 glucose, 10 HEPES, and 2 MgCl_2_, and pH 7.3 adjusted with NaOH. The PSS containing Ca^2+^ for UBSM contractility studies was prepared daily and contained the following (mM): 119 NaCl, 4.7 KCl, 24 NaHCO_3_, 1.2 KH_2_PO_4_, 2.5 CaCl_2_, 1.2 MgCl_2_, 11 glucose, and aerated with 95% O_2_‐5% CO_2_ to maintain a pH of 7.4. Glibenclamide (catalog no. G0639) and 9‐phenanthrol (catalog no. 211281) were purchased from Millipore‐Sigma and prepared as stock solutions of 30–100 mM in dimethyl sulfoxide (DMSO; Millipore‐Sigma, catalog no. D8418). Subsequent serial dilutions were made in PSS and tested at various increasing concentrations (cumulatively) as specified.

### 2.5 Data analyses and statistics

Isometric UBSM tension recordings were performed using MyoMed software (Med Associates). MiniAnalysis software (Synaptosoft) was utilized for UBSM phasic contraction parameter analysis including amplitude, muscle force integral (integrated area under contraction curve), duration (calculated as half‐width), frequency, and muscle tone over the final 5 min before the addition of the next higher concentration of 9‐phenanthrol or glibenclamide. GraphPad Prism 8.0 software (GraphPad Software) and Excel (Microsoft) were used for statistical analysis including a test for normal distribution, ANOVA, or Student's *t*‐test as appropriate. We present the data as normalized values to the control prior to the addition of any compound. In addition, we summarize the raw data for the controls in UBSM‐J and UBSM‐A of spontaneous and 20 mM K^+^‐induced contractions, respectively, in Suporting Information (https://doi.org/10.6084/m9.figshare.13611383.v1). Microsoft PowerPoint 2016 was used for data illustration. Normally distributed data are summarized as means ± SEM, while data not distributed normally are summarized as medians with 25th and 75th percentiles given; *n* = number of strips mounted for isometric UBSM tissue tension recordings; *N *= number of guinea pigs used. For this study, a *p* < 0.05 (two‐tailed for all) was considered statistically significant. Data sets were compared using the two‐way ANOVA followed by the post hoc Sidak multiple comparison test or Student's *t*‐test when appropriate.

## 3 RESULTS

### 3.1 Total, surface, and intracellular TRPM4 channel protein expression are significantly decreased in adult (UBSM‐A) compared to juvenile UBSM (UBSM‐J), while the ratios of surface‐to‐intracellular in both UBSM age groups remain unchanged

**FIGURE 1:**
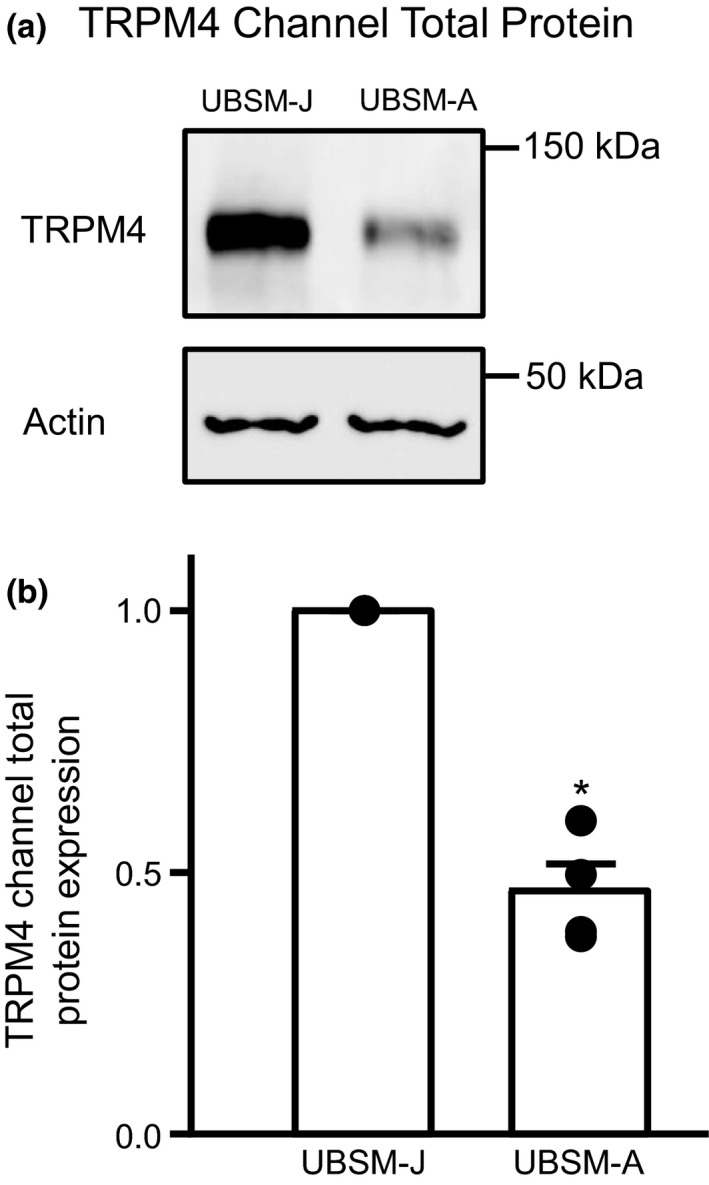
TRPM4 channel total protein is reduced in UBSM‐A compared to UBSM‐J. (a) Representative Western blots show that the molecular weight of TRPM4 was 130 kDa in both UBSM‐J and UBSM‐A. (b) TRPM4 channel total protein expression (shown as fold‐change normalized to UBSM‐J and actin) was reduced by ~50% in UBSM‐A compared to UBSM‐J (*N* = 4 for both groups, *p < 0.05).

To compare the protein level of TRPM4 channels in UBSM‐A and UBSM‐J, we first performed Western blotting on homogenates under the conditions reflecting total protein expression. The same molecular weight of guinea pig TRPM4 protein of ∼130 kDa was detected in UBSM‐A and UBSM‐J (Figure 1). Overall, the TRPM4 channel protein was significantly reduced by ~50% in UBSM‐A as compared to UBSM‐J with normalization to both UBSM‐J and β‐actin (Figure 1). These findings indicate a reduction of total TRPM4 protein in UBSM‐A.

**FIGURE 2:**
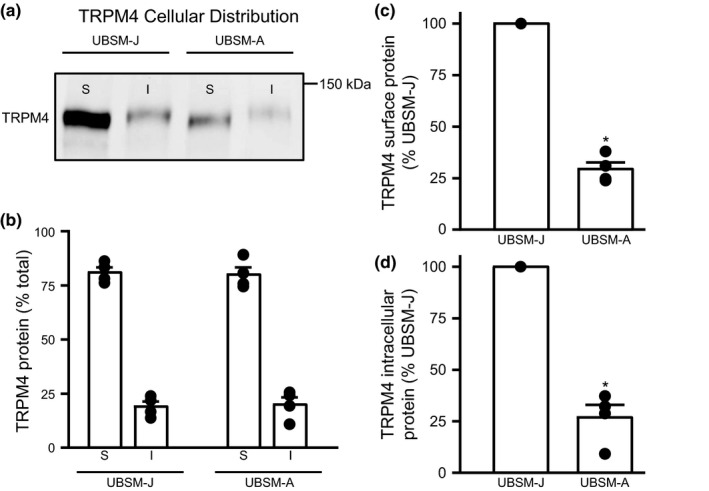
Cellular distribution of TRPM4 channel protein remains unchanged between UBSM‐J and UBSM‐A. (a) Representative Western blot illustrates the cellular distribution of TRPM4 protein in UBSM. (b) Both UBSM‐J and UBSM‐A indicated that 80% of TRPM4 protein was located in the cell surface fraction compared to the intracellular fraction. (c) Surface TRPM4 protein expression was reduced by ~70% in UBSM‐A compared to UBSM‐J (*N* = 4 both groups, *p < 0.05). (d) UBSM‐A showed a ~70% reduction in intracellular TRPM4 protein compared to UBSM‐J(*N* = 4 for both groups, *p < 0.05). S, surface fraction; I, intracellular fraction.

To further investigate if the decrease in total TRPM4 protein changed the cellular localization of the channel in UBSM‐A and UBSM‐J, we used the surface biotinylation procedure. Cell surface biotinylation of smooth muscle tissue has been well characterized for arterial smooth muscle previously (Hasan et al., 2019; Leo et al., 2015). The same protocol with minor adjustments was used to examine TRPM4 channel localization in UBSM‐J and UBSM‐A. Surface biotinylation showed that ∼80% of TRPM4 protein was located in the plasma membrane in both UBSM‐J and UBSM‐A (Figure 2). Even though the overall cellular distribution of TRPM4 did not change in UBSM‐A, for both surface and intracellular TRPM4 channel protein there was a ~70% decrease compared to UBSM‐J (Figure 2). Thus, these findings demonstrate that there were significant reductions of the total, plasma membrane, and intracellular fractions for the TRPM4 channel protein in UBSM‐A compared to UBSM‐J.

### 3.2 TRPM4 channel inhibitor 9‐phenanthrol attenuates UBSM‐J spontaneous and 20 mM KCl‐induced phasic contractions with increased potency compared to UBSM‐A responses

**FIGURE 3:**
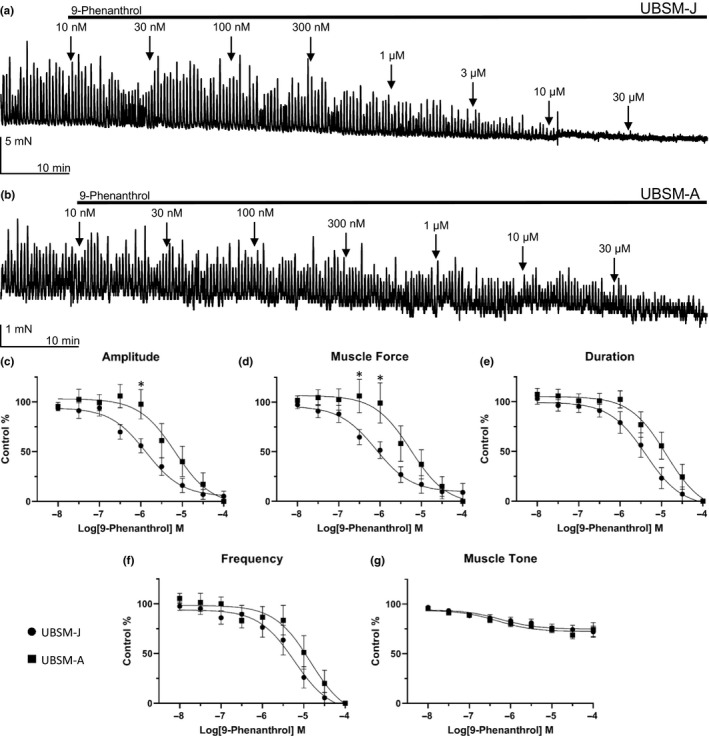
9‐Phenanthrol inhibits spontaneous phasic contractions with lower potency in UBSM‐A compared to UBSM‐J. (a) Representative isometric tension recording from a UBSM‐J isolated strip illustrating the effect of cumulative applications of 9‐phenanthrol (10 nM–30 μM) on UBSM spontaneous phasic contractions. (b) Representative isometric tension recording from a UBSM‐A isolated strip illustrating the effect of cumulative applications of 9‐phenanthrol (30 nM–30 μM) on UBSM spontaneous phasic contractions. (c–g) Cumulative concentration‐response curves for the inhibitory effects of 9‐phenanthrol (10 nM–100 μM) on UBSM phasic contraction amplitude (c), muscle force (d), duration (e), frequency (f), and muscle tone (g), (UBSM‐J: *n* = 6–13, *N* = 4; UBSM‐A: *n* = 4–12, *N* = 7–9). The two‐way ANOVA analysis revealed a statistically significant difference in phasic contraction amplitude and muscle force between UBSM‐J and UBSM‐A [UBSM‐J vs UBSM‐A, amplitude: *F*(1,19) = 4.803, *p* < 0.05; muscle force: *F*(1,18) = 4.746, *p* < 0.05]; asterisks denote statistically significant differences for UBSM‐J vs UBSM‐A (two‐way ANOVA, post hoc Sidak multiple comparison test, *p < 0.05).

For the initial series of experiments, responses to cumulative additions of 9‐phenanthrol, a TRPM4 channel inhibitor, were evaluated for UBSM‐J and UBSM‐A on spontaneous phasic contractions and are illustrated in Figure 3. 9‐Phenanthrol caused a concentration‐dependent reduction in all parameters for spontaneous phasic contractions of both UBSM‐A and UBSM‐J strips. The IC_50_ values for phasic contraction amplitude and muscle force were both at least five times lower in the UBSM‐J group, 1.3 and 0.8 μM, respectively, than for UBSM‐A, 6.7 and 5.5 μM, respectively. At the highest concentration of 100 µM, the maximum efficacies for 9‐phenanthrol on spontaneous phasic UBSM contractions were equivalent for UBSM‐A and UBSM‐J. In UBSM‐A contraction amplitude, muscle force, duration, and frequency were completely inhibited (100%) by 100 µM 9‐phenanthrol, and similarly, UBSM‐J showed either full or near‐complete maximum inhibition (91.0–100%) for these contraction parameters (Table 1). Both the contraction amplitude and muscle force showed statistically significant differences between the groups (UBSM‐J vs UBSM‐A, amplitude: *F*(1,19) = 4.803, *p* < 0.05; muscle force: *F*(1,18) = 4.746, *p* < 0.05, two‐way ANOVA).

**TABLE 1:** Comparative summary of potency and maximum efficacy values for inhibitory effects of 9‐phenanthrol on spontaneous phasic contractions in UBSM‐A and UBSM‐J. 9‐Phenanthrol inhibited spontaneous phasic contractions in UBSM‐J with greater potency on contraction amplitude, muscle force, duration, and frequency than in UBSM‐A. CI, confidence interval.

	UBSM‐J	UBSM‐A
Phasic contraction parameter	IC_50_, mean (95% CI) Max inhibition, mean ± SEM (*n* = 6–13, *N* = 4)	IC_50_, mean (95% CI) Max inhibition, mean ± SEM (*n* = 4–12, *N* = 7–9)
Amplitude	1.3 (0.7–2.6) μM 94.8 ± 5.2%	6.7 (2.4–20.5) μM 100.0 ± 0.0%
Muscle force	0.8 (0.4–1.6) μM 91.0 ± 9.1%	5.5 (1.9–17.8) μM 100.0 ± 0.0%
Duration	4.1 (2.1–8.2) μM 100.0 ± 0.0%	12.8 (4.9–3.5) μM 100.0 ± 0.0%
Frequency	5.9 (2.8–12.5) μM 100.0 ± 0.0%	14.4 (4.8–45.0) μM 100.0 ± 0.0%
Muscle tone	0.6 (0.07–4.6) μM 27.8 ± 5.7%	0.5 (0.1–2.0) μM 25.6 ± 7.0%

**FIGURE 4:**
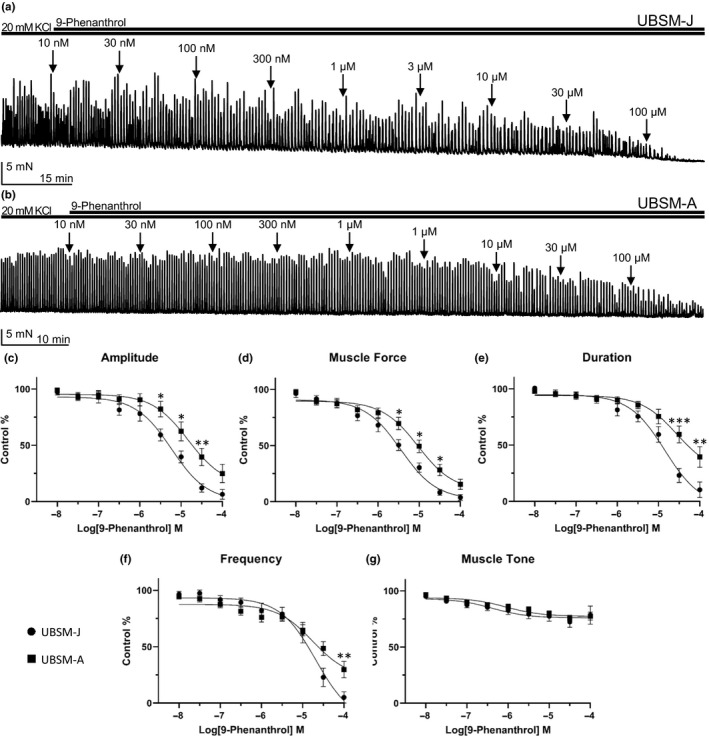
9‐Phenanthrol inhibits 20 mM KCl‐induced phasic contractions with lower potency and maximum efficacy in UBSM‐A compared to UBSM‐J. (a) Representative isometric tension recording from a UBSM‐J isolated strip illustrating the effect of cumulative applications of 9‐phenanthrol (10 nM–100 μM) on UBSM 20 mM KCl‐induced phasic contractions. (b) Representative isometric tension recording from a UBSM‐A isolated strip illustrating the effect of cumulative applications of 9‐phenanthrol (10 nM–100 μM) on UBSM 20 mM KCl‐induced phasic contractions. (c–g) Cumulative concentration‐response curves for the inhibitory effects of 9‐phenanthrol (10 nM–100 μM) on UBSM phasic contraction amplitude (c), muscle force (d), duration (e), frequency (f), and muscle tone (g), (UBSM‐J: *n* = 10–23, *N* = 8–10; UBSM‐A: *n* = 19–33, *N* = 10–13). The two‐way ANOVA analysis revealed a statistically significant difference in phasic contraction amplitude, muscle force, and duration between UBSM‐J and UBSM‐A [UBSM‐J vs UBSM‐A, amplitude: *F*(1,45) = 6.054, *p* < 0.05; muscle force: *F*(1,50)=4.802, *p* < 0.05; duration: *F*(1,42) = 4.768, *p* < 0.05]; asterisks denote statistically significant differences for UBSM‐J vs UBSM‐A (two‐way ANOVA, post hoc Sidak multiple comparison test, *p < 0.05, **p < 0.01, ***p < 0.001).

The effect of the TRPM4 channel inhibitor 9‐phenanthrol was then investigated on UBSM contractility where 20 mM KCl was used to induce sustained membrane depolarization, which increases phasic and tonic contractions in isolated UBSM strips. Cumulative concentration‐response curves to 9‐phenanthrol on 20 mM KCl‐induced phasic contractions in UBSM‐J and UBSM‐A are summarized in Figure 4. In both UBSM‐J and UBSM‐A, 9‐phenanthrol caused a concentration‐dependent reduction in all contraction parameters for 20 mM KCl‐induced phasic UBSM contractions. In UBSM‐J, the inhibition potencies for 9‐phenanthrol on phasic contraction amplitude, muscle force, duration, and muscle tone were approximately two‐fold higher in comparison to UBSM‐A. Specifically, the IC_50_ values for amplitude and muscle force in UBSM‐J were 5.9 and 3.6 μM, respectively, compared to 14.8 and 9.3 μM for UBSM‐A. Additionally, UBSM‐J displayed higher maximum efficacies compared to UBSM‐A on amplitude (93.6% vs 75.1%), muscle force (96.3% vs 84.5%), duration (89.7% vs 60.9%), and frequency (95.0% vs 70.3%) (Table 2). There was a statistically significant difference between UBSM‐J and UBSM‐A for phasic contraction amplitude, muscle force, and duration (UBSM‐J vs UBSM‐A, amplitude: *F*(1,45) = 6.054, *p* < 0.05; muscle force: *F*(1,50) = 4.802, *p* < 0.05; duration: *F*(1,42) = 4.768, *p* < 0.05, two‐way ANOVA). Additionally, we also present the raw data comparing UBSM‐J and UBSM‐A for both spontaneous and 20 mM KCl‐induced contractions. The data analysis did not show any statistical differences between UBSM‐J and UBSM‐A on any parameter including frequency for spontaneous or 20 mM KCl‐induced contractions (Figures S3 and S4, https://doi.org/10.6084/m9.figshare.13611383.v1). Collectively, 9‐phenanthrol had increased potency on UBSM‐J compared to UBSM‐A for both spontaneous and 20 mM KCl‐induced phasic contractions. 9‐Phenanthrol also exhibited an increased effect in UBSM‐J to concentrations lower than 0.3 µM for 20 mM KCl‐induced phasic contractions, while spontaneous phasic contractions showed an increased effect in UBSM‐J for concentrations between 0.1 and 0.3 µM.

**TABLE 2:** Comparative summary of potency and maximum efficacy values for inhibitory effects of 9‐phenanthrol on 20 mM KCl‐induced phasic contractions in UBSM‐A and UBSM‐J. 9‐Phenanthrol inhibited 20 mM KCl‐induced phasic contractions in UBSM‐J with greater potency on contraction amplitude, muscle force, duration, and muscle tone than in UBSM‐A. CI, confidence interval.

	UBSM‐J	UBSM‐A
Phasic contraction parameter	IC_50_, Mean (95% CI) Max inhibition, mean ± SEM (*n* = 10–23, *N* = 8–10)	IC_50_, Mean (95% CI) Max inhibition, mean ± SEM (*n* = 19–33, *N* = 10–13)
Amplitude	5.9 (3.5–8.2) μM 93.6 ± 4.3%	14.8 (7.1–30.8) μM 75.1 ± 8.0%
Muscle force	3.6 (2.2–5.2) μM 96.3 ± 2.5%	9.3 (5.5–15.9) μM 84.5 ± 4.6%
Duration	14.3 (8.1–24.8) μM 89.7 ± 7.0%	27.9 (1.1–83.6) μM 60.6 ± 9.1%
Frequency	20.8 (10.9–40.3) μM 95.0 ± 5.0%	17.4 (7.2–42.8) μM 70.3 ± 7.3%
Muscle tone	0.4 (0.03–3.9) μM 21.5 ± 8.1%	0.9 (0.2–4.3) μM 22.5 ± 3.6%

### 3.3 TRPM4 channel inhibitor glibenclamide attenuates UBSM‐J spontaneous and 20 mM KCl‐induced phasic contractions with increased potency compared to UBSM‐A

**FIGURE 5:**
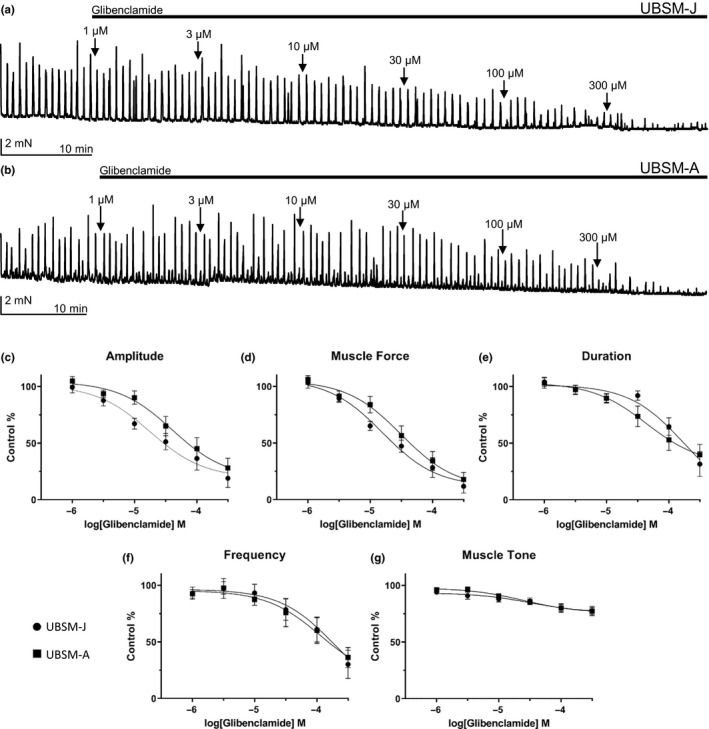
Glibenclamide inhibits spontaneous phasic contractions with lower potency in UBSM‐A compared to UBSM‐J. (a) Representative isometric tension recording from a UBSM‐J isolated strip illustrating the effect of cumulative applications of glibenclamide (1–300 μM) on UBSM 20 mM KCl‐induced phasic contractions. (b) Representative isometric tension recording from a UBSM‐A isolated strip illustrating the effect of cumulative applications of glibenclamide (1–300 μM) on UBSM 20 mM KCl‐induced phasic contractions. (c–g) Cumulative concentration‐response curves for the inhibitory effects of glibenclamide (1–300 μM) on UBSM phasic contraction amplitude (c), muscle force (d), duration (e), frequency (f), and muscle tone (g), (UBSM‐J: *n* = 9–10, *N* = 5–6; UBSM‐A: *n* = 10, *N* = 6).

**FIGURE 6:**
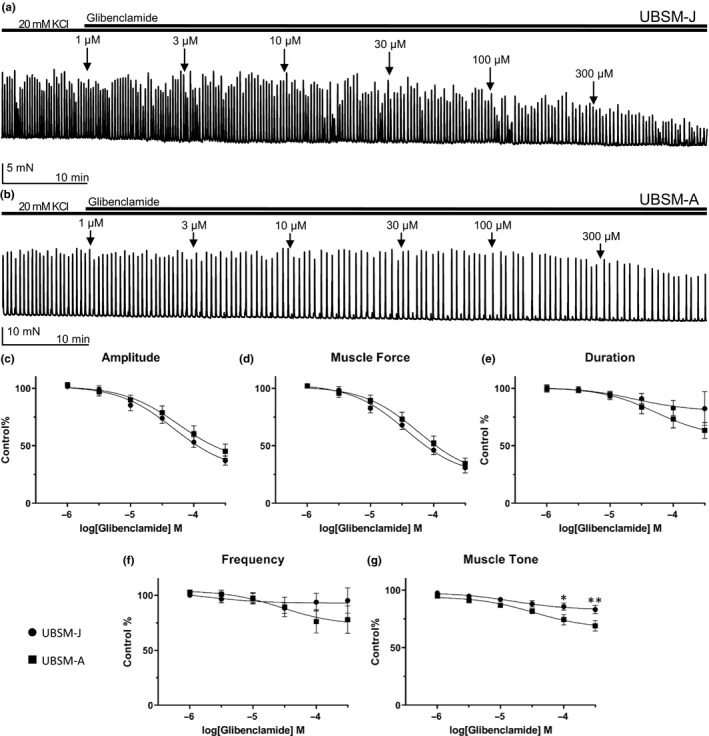
Glibenclamide inhibits 20 mM KCl‐induced phasic contractions with lower potency in UBSM‐A compared to UBSM‐J. (a) Representative isometric tension recording from a UBSM‐J isolated strip illustrating the effect of cumulative applications of glibenclamide (1–300 μM) on UBSM 20 mM KCl‐induced phasic contractions. (b) Representative isometric tension recording from a UBSM‐A isolated strip illustrating the effect of cumulative applications of glibenclamide (1–300 μM) on UBSM 20 mM KCl‐induced phasic contractions. (c–g) Cumulative concentration‐response curves for the inhibitory effects of glibenclamide (1–300 μM) on UBSM phasic contraction amplitude (c), muscle force (d), duration (e), frequency (f), and muscle tone (g), (UBSM‐J: *n* = 27–29, *N* = 10; UBSM‐A: *n* = 15–21, *N* = 7). The two‐way ANOVA analysis revealed a statistically significant difference in phasic contraction muscle force between UBSM‐J and UBSM‐A [UBSM‐J vs UBSM‐A, amplitude: *F*(1,45) = 6.054, *p* < 0.05; muscle force: *F*(1,47) = 4.811, *p* < 0.05]; asterisks denote statistically significant differences for UBSM‐J vs UBSM‐A (two‐way ANOVA, post hoc Sidak multiple comparison test, *p < 0.05, **p < 0.01).

The effects of glibenclamide on UBSM contractility were examined on both spontaneous phasic and 20 mM KCl‐induced phasic contractions. Cumulative applications of increasing concentrations of glibenclamide effectively attenuated spontaneous and 20 mM KCl‐induced phasic contractions in UBSM‐J and UBSM‐A strips. For spontaneous contractions, IC_50_ values for inhibition of UBSM‐J phasic contraction amplitude (17.6 µM) and muscle force (15.8 µM) were approximately 50% lower than that of UBSM‐A (40.6 and 31.4 µM, respectively) (Figure 5, Table 3). Contraction amplitude was the only parameter that showed a difference in maximum efficacy between the two groups (UBSM‐J: 81.1% vs UBSM‐A: 72.0%) (Table 3). Overall, two‐way ANOVA analysis did not reveal any statistically significant differences between UBSM‐J and UBSM‐A spontaneous contractions for all of the parameters analyzed (Figure 6). In the case of 20 mM KCl‐induced phasic contractions, glibenclamide showed increased potency for amplitude, muscle force, duration, frequency, and muscle tone in UBSM‐J. Additionally, the effect of glibenclamide showed a statistically significant difference between the two groups on muscle tone (UBSM‐J vs UBSM‐A, muscle tone: *F*(1,47) = 4.811, *p* < 0.05, two‐way ANOVA).

**TABLE 3:** Comparative summary of potency and maximum efficacy values for inhibitory effects of glibenclamide on spontaneous phasic contractions in UBSM‐A and UBSM‐J. Glibenclamide inhibited UBSM‐J spontaneous phasic contractions with greater potency on contraction amplitude and muscle force than in UBSM‐A. CI, confidence interval.

	UBSM‐J	UBSM‐A
Phasic contraction parameter	IC_50_, Mean (95% CI) Max inhibition, mean ± SEM (*n* = 9–10, *N* = 5–6)	IC_50_, Mean (95% CI) Max inhibition, mean ±SEM (*n* = 10, *N* = 6)
Amplitude	17.6 (6.9–45.2) μM 81.1 ± 8.1%	40.6 (15.4–106.8) μM 72.0 ± 8.7%
Muscle force	15.8 (8.2–30.6) μM 88.2 ± 7.4%	31.4 (14.2–69.1) μM 82.1 ± 6.1%
Duration	169.4 (45.7–627.9) μM 68.4 ± 11.0%	43.9 (13.7–140.6) μM 60.1 ± 9.1%
Frequency	187.3 (21.8–161.3) μM 69.8 ± 12.5%	113.9 (20.0–647.8) μM 63.6 ± 8.9%
Muscle tone	32.0 (4.2–245.3) μM 22.6 ± 4.0%	25.4 (8.8–73.7) μM 22.8 ± 3.0%

## 4 DISCUSSION

Here, we report on novel findings that TRPM4 channels regulate UBSM function by demonstrating the following: (a) a 50–70% decrease in the normalized total expression of TRPM4 channels in UBSM‐A versus UBSM‐J, (b) predominant plasma membrane expression of TRPM4 channels and a similar ratio of surface‐to‐intracellular expression in both age groups consistent with a role of TRPM4 channels in regulating UBSM cell excitability and contractility, and (c) reduced effectiveness of the TRPM4 channel blocker 9‐phenanthrol in UBSM‐A compared to UBSM‐J on inhibiting spontaneous and 20 mM KCl‐induced phasic contractions. TRPM4 channels, hence, display an age‐dependent (juvenile vs adult) expression profile correlating with the pharmacologically differential effects of 9‐phenanthrol on contractility in the two age groups.

Ion channels that determine smooth muscle excitability need to be present on the plasma membrane to exert their regulatory control. Cellular localization analyses of ion channel subunits have been done previously in arterial smooth muscle (Hasan et al., 2019; Leo & Jaggar, 2017). However, there are no reports so far on UBSM ion channel trafficking. Here, we detail a novel finding that identified TRPM4 channel total expression and cellular protein distribution (surface vs intracellular) in adult and juvenile UBSM. We observed that total, surface, and intracellular TRPM4 channel protein expression were decreased in UBSM‐A compared to UBSM‐J, while the ratios of surface‐to‐intracellular in both UBSM age groups remain unchanged. This finding reveals an age‐dependent decrease in TRPM4 channel expression that could be attributed to a posttranscriptional or posttranslational regulatory mechanism. Interestingly, surface or plasma membrane expression accounted for ∼75–80% of total cellular expression in both UBSM‐J and UBSM‐A. This indicates that in UBSM, TRPM4 channel trafficking mechanisms provide optimal delivery of TRPM4 channels to the plasma membrane.

Overall, we detected robust TRPM4 protein expression in UBSM‐J. The band size of the TRPM4 channel protein was ∼130 kDa, which corresponds to the computed molecular weight. Similar band size was reported in UBSM lysates from human (Hristov et al., 2016) and mouse bladder (Kullmann et al., 2018). To effectively control smooth muscle excitability, TRPM4 channels are expected to be localized to the plasma membrane rather than intracellularly. This has been reported in cerebral artery smooth muscle where ∼65% of total TRPM4 protein was surface localized (Crnich et al., 2010). Here, we established that in guinea pig UBSM, ∼80% of total TRPM4 protein is surface localized. This indicates that TRPM4 channels play an essential role in UBSM perhaps even more pronounced than in arterial smooth muscle. Intriguingly, the expression of TRPM4 mRNA in UBSM exceeded that of in the arteries (Parajuli et al., 2013) supporting differential roles of TRPM4 channels in regulating function in different smooth muscle tissues. In arterial smooth muscle, protein kinase C (PKC) mediates the acute trafficking of TRPM4 channels to the plasma membrane increasing the surface expression up to ∼80% (Crnich et al., 2010). Whether PKC also regulates TRPM4 channel trafficking in UBSM remains unknown. Given the already high level of plasma membrane expression, ∼80% of total cellular TRPM4 protein, PKC is unlikely to increase channel trafficking effectively. In UBSM, however, the expression of TRPM4 may be changed under pathophysiological conditions. In mice that underwent spinal cord injury, there was a phasic rise and fall in TRPM4 protein over a period of 28 days (Kullmann et al., 2018). These studies indicate that TRPM4 expression in smooth muscle is prone to be modulated both acutely and long term. Our results show that there was a significant decrease in total UBSM TRPM4 channel protein expression in UBSM‐A. Consistent with this, there was a decrease in surface localized TRPM4 protein in UBSM‐A too. Collectively, these results suggest a reduction of TRPM4 channel function in adult UBSM.

To investigate the TRPM4 channel functional consequences on UBSM, we examined the inhibitory effects of two structurally distinct TRPM4 channel inhibitors, 9‐phenanthrol and glibenclamide, on UBSM phasic contractility. The results indicated a decrease for both spontaneous and 20 mM KCl‐induced UBSM‐J phasic contractions in response to 9‐phenanthrol in comparison to the effect in UBSM‐J. Indeed, the potency for 9‐phenanthrol on spontaneous phasic UBSM contractions was five‐ to seven‐fold higher for UBSM‐J than UBSM‐A for contraction amplitude and muscle force, and the potency for glibenclamide on the spontaneous phasic UBSM contractions was twofold higher for UBSM‐J than UBSM‐A for amplitude and muscle force as well. For 20 mM KCl‐induced UBSM contractions, 9‐phenanthrol showed twofold higher potency for the UBSM‐J compared to the UBSM‐A for amplitude and muscle force, while glibenclamide showed comparable potencies for both groups. Additionally, concentration responses to 9‐phenanthrol on spontaneous phasic UBSM contractions comparing UBSM‐J and UBSM‐A responses were significantly different for amplitude and muscle force (Figure 3), while glibenclamide did not show a statistical difference for the two groups (Figure 5). Thus, in UBSM‐A, the reduction of total TRPM4 protein correlated with the reduced efficacy of the TRPM4 channel inhibitor 9‐phenanthrol on UBSM contractility. This finding provides additional evidence for the differential, age‐dependent role of TRPM4 channels in UBSM function. However, an analysis of the raw data for both spontaneous and 20 mM KCl‐induced phasic contractions did not show any statistical difference between UBSM‐J and UBSM‐A (Figures S3 and S4, https://doi.org/10.6084/m9.figshare.13611383.v1). This is consistent with a previously mentioned study that reported no difference in maximum induced responses from EFS for the UBSM of adult humans of varying ages (Fry et al., 2011). It is possible that TRPM4 channel protein expression decreases as a compensatory mechanism in order to maintain a physiological level of excitability of the UBSM as guinea pigs mature from juveniles to adults.

The inhibitory effects of 9‐phenanthrol on spontaneous and 20 mM KCl‐induced phasic contractions observed here for UBSM‐J and UBSM‐A are consistent with prior findings for guinea pig, rat, and human UBSM (Demion et al., 2007; Grand et al., 2008; Malysz et al., 2020; Ozhathil et al., 2018). In agreement with our previous report (Malysz et al., 2020), 9‐phenanthrol showed higher effectiveness than glibenclamide in attenuating spontaneous and 20 mM KCl contractions (Tables 1, 2, 3, 4). Given the reported higher potency of 9‐phenanthrol (20 µM) than glibenclamide (40 µM) on recombinant TRPM4 channels, more robust effects of 9‐phenanthrol on UBSM contractility were expected and observed. Indeed, 9‐phenanthrol displayed much stronger effects than glibenclamide on UBSM phasic contractions regardless of the age group tested or contraction protocol examined. However, differences in the maximum inhibition profiles for the two TRPM4 inhibitors cannot be easily explained via a simple antagonism mechanism. For example, spontaneous phasic contractions were nearly completely inhibited by 9‐phenanthrol (at 100 µM, Figure 3), whereas the maximum concentration of glibenclamide (300 µM) caused incomplete inhibition (Figure 5). The reasons for the differential pharmacological effects of 9‐phenanthrol and glibenclamide are yet to be elucidated. Previously, we proposed that 9‐phenanthrol and glibenclamide utilized differential inhibitory mechanisms on guinea pig TRPM4 channels resulting in full and partial channel blockage, respectively (Malysz et al., 2020). In support of this concept, glibenclamide only partially inhibited 9‐phenanthrol‐sensitive voltage step‐induced cation currents in freshly isolated guinea pig UBSM cells (Malysz et al., 2020). One possible explanation for the differential effects of 9‐phenanthrol and glibenclamide is the involvement of SUR‐TRPM4 channel complexes in UBSM. While under certain experimental conditions, especially pathological such as ischemia in the brain, SUR‐TRPM4 co‐assemble to form functional complexes where glibenclamide via direct interaction with the SUR inhibits the complex activity (Malysz et al., 2020; Mehta et al., 2015). In UBSM, however, we found no evidence for SUR‐TRPM4 complexes (Malysz et al., 2020). Diazoxide, an activator of SUR‐TRPM4 complexes, did not increase UBSM whole‐cell cation currents as would be expected if these complexes were present and regulating excitability. An alternative explanation might be that the two compounds differentially target channels or receptors other than TRPM4 to inhibit UBSM contractility. Indeed, 9‐phenanthrol and glibenclamide were shown to affect targets other than TRPM4 at similar concentrations to those active on TRPM4 channels including cystic fibrosis transmembrane conductance regulator (CFTR) channels, other ATP binding cassette (ABC) proteins, and voltage‐gated K_V_ channels for glibenclamide (Melin et al., 2007; Payen et al., 2001; Schaffer et al., 1999) as well as L‐type Ca_V_, Ca^2+^‐activated TMEM16A/Ano1, and intermediate conductance Ca^2^^+^‐activated K^+^ channels for 9‐phenanthrol (Burris et al., 2015; Simard et al., 2012; Veress et al., 2018).

**TABLE 4:** Comparative summary of potency and maximum efficacy values for inhibitory effects of glibenclamide on 20 mM KCl‐induced phasic contractions in UBSM‐A and UBSM‐J. Glibenclamide inhibited UBSM‐J 20 mM KCl‐induced phasic UBSM contractions with greater potency on contraction amplitude, muscle force, duration, frequency, and muscle tone than UBSM‐A. CI, confidence interval.

	UBSM‐J	UBSM‐A
Phasic contraction parameter	IC_50_, Mean (95% CI) Max inhibition, mean ± SEM (*n* = 27–29, *N* = 10)	IC_50_, Mean (95% CI) Max inhibition, mean ± SEM (*n* = 15–21, *N* = 7)
Amplitude	48.1 (24.9–92.9) μM 62.9 ± 4.0%	57.0 (21.5–150.9) μM 54.8 ± 6.2%
Muscle force	36.6 (21.7–61.8) μM 69.0 ± 4.5%	56.3 (26.4–119.7) μM 65.4 ± 4.7%
Duration	24.7 (0.5–1106) μM 17.6 ± 14.6%	53.7 (11.8–243.6) μM 36.7 ± 7.0%
Frequency	2.0 μM (0.1 nM–0.3 M) 4.8 ± 11.5%	28.6 (1.7–484.8) μM 22.1 ± 12.4%
Muscle tone	15.4 (2.4–97.5) μM 16.9 ± 3.6%	33.4 (9.8–113.7) μM 31.0 ± 4.6%

Our novel findings of the reduction in the expression of TRPM4 protein in UBSM‐A compared to UBSM‐J add to the mechanisms already shown to change during developmental or maturation stages. For example, a study comparing UBSM responses of fetal and adult sheep revealed altered muscarinic receptor sensitivity as the older group displayed higher potency for the agonist carbachol. Additionally, 10 µM α,β‐methylene ATP, a purinergic agonist, elicited significantly greater Ca^2+^ transient currents in adult UBSM myocytes compared to their fetal counterpart suggesting greater P2X receptor expression in the adult (Wu et al., 2006). In line with previously mentioned studies, adult porcine UBSM (>40 weeks), when compared to juvenile UBSM (8–12 weeks), showed a higher number of muscarinic receptor binding sites as well as an increased potency to carbachol for stimulation of contractility (Wuest et al., 2008). In another study, investigators determined via PCR that there was a lower expression of purinergic receptors in human fetal bladder compared to adults (O'Reilly et al., 2001). Additionally, younger animals have shown greater UBSM contractility than matured animals. The UBSM of newborn rats has been observed to show significantly increased contractile responses to KCl, carbachol, and ATP compared to the responses of 1‐month‐old and young adult (4‐month‐olds; Tugay et al., 2003). Relevant here, muscarinic receptor signaling can be influenced by TRPM4 modulation as well. Several publications have shown that the application of inhibitors of TRPM4 channels to phasic contractions induced by the muscarinic agonist carbachol leads to a decrease in contractility (Hristov et al., 2016; Smith, Hristov, et al., 2013; Smith, Parajuli, et al., 2013). Further, TRPM4 channels have been reported to colocalize in close proximity with inositol 1,4,5‐triphosphate receptors (IP_3_Rs) in human UBSM cells (Provence et al., 2017). Sarcoplasmic reticulum IP_3_Rs have been shown to regulate TRPM4 channel‐mediated transient inward cation currents in human UBSM cells through the Ca^2+^ release mechanism (Provence et al., 2017). Thus, muscarinic receptor stimulation can also lead to downstream activation of TRPM4 channels via intracellular Ca^2+^ increase signaling. However, how TRPM4 channel expression, function, and regulation change in human UBSM during different life stages, and particularly in the adult and aging population, remain unexplored.

In summary, this is the first study to show subcellular localization and distribution profiles of TRPM4 channels in UBSM of two age groups, juveniles and adults. Based on an experimental animal model, we revealed a novel age‐dependent role of TRPM4 channels in UBSM function. We reveal that total, surface, and intracellular TRPM4 channel protein were decreased in adult compared to juvenile UBSM. In both age groups, regardless of the overall reduced total TRPM4 protein expression in UBSM‐A, cell surface TRPM4 protein expression (~80%) predominated over its intracellular fraction (~20%), revealing preserved channel trafficking mechanisms toward the cell membrane. Since both age groups displayed similar very high ratios of surface‐to‐intracellular TRPM4 channel expression, trafficking mechanisms in UBSM promote preferential robust expression of TRPM4 channels to the plasma membrane as would be expected for an ion channel that is critically important for regulating UBSM cell function. Further, we showed that 9‐phenanthrol exerted a reduced inhibitory effect on UBSM‐A, both spontaneous and 20 mM KCl‐induced phasic UBSM contractions, where the expression of the total TRPM4 protein was reduced compared to UBSM‐J. Collectively, our data reveal that reduced expression of TRPM4 channel protein with age alters guinea pig UBSM function. Future comparative studies on human UBSM isolated from patients with or without OAB from various age groups will provide insights as to if TRPM4 channel dysfunction is a contributing factor to OAB that increases with age.

## DISCLOSURES

No conflict of interest, financial, or otherwise is declared by the authors.

## AUTHOR CONTRIBUTIONS

S.E.M. and M.D.L. performed experiments; S.E.M., M.D.L., and J.M. analyzed data; S.E.M., M.D.L., J.M., and G.V.P. interpreted results of experiments; S.E.M., M.D.L., J.M., and G.V.P. prepared figures; S.E.M., M.D.L., J.M., and G.V.P. drafted manuscript; S.E.M., M.D.L., J.M., and G.V.P. edited and revised manuscript; S.E.M., M.D.L., J.M., and G.V.P. approved final version of manuscript.

## Supporting information



Figs S1‐S2
