# Supplementary material for: Age‐dependent decrease in TRPM4 channel expression but not trafficking alters urinary bladder smooth muscle contractility
Source: Physiol Rep. 2021 Feb 24;9(4):e14754. doi: 10.14814/phy2.14754 (PMC7903938; doi:10.14814/phy2.14754)
Supplement: Supplementary file 1 — Figs S1‐S2 [file PHY2-9-e14754-s001.pdf]

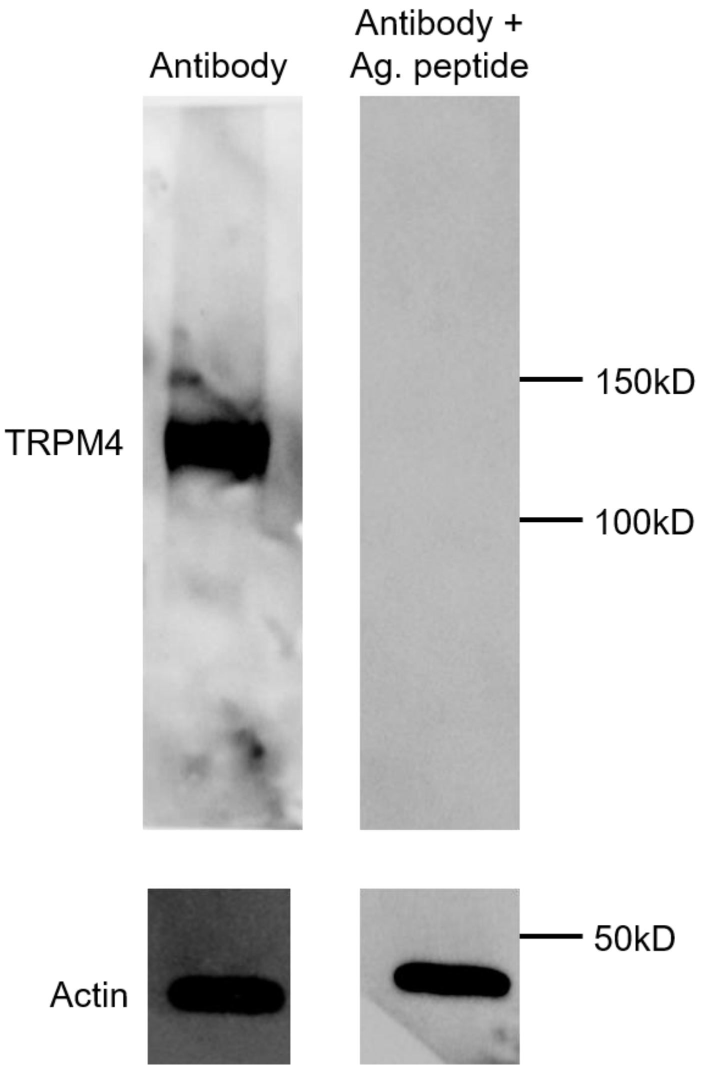

Suppl. Fig. 1. *Validation of the TRPM4 antibody.* Left, Representative blot showing total TRPM4 protein (guinea pig urinary bladder smooth muscle) after addition of rabbit polyclonal anti-TRPM4 antibody (Aviva Systems Biology, Cat#ARP35268_P050). Right, Same blot after strip with Restore™ PLUS Western Blot Stripping Buffer (Thermo Scientific Inc.) and re-probed with pre-mix of the rabbit polyclonal anti-TRPM4 antibody + antigenic peptide (Aviva Systems Biology, Cat#AAP35268).

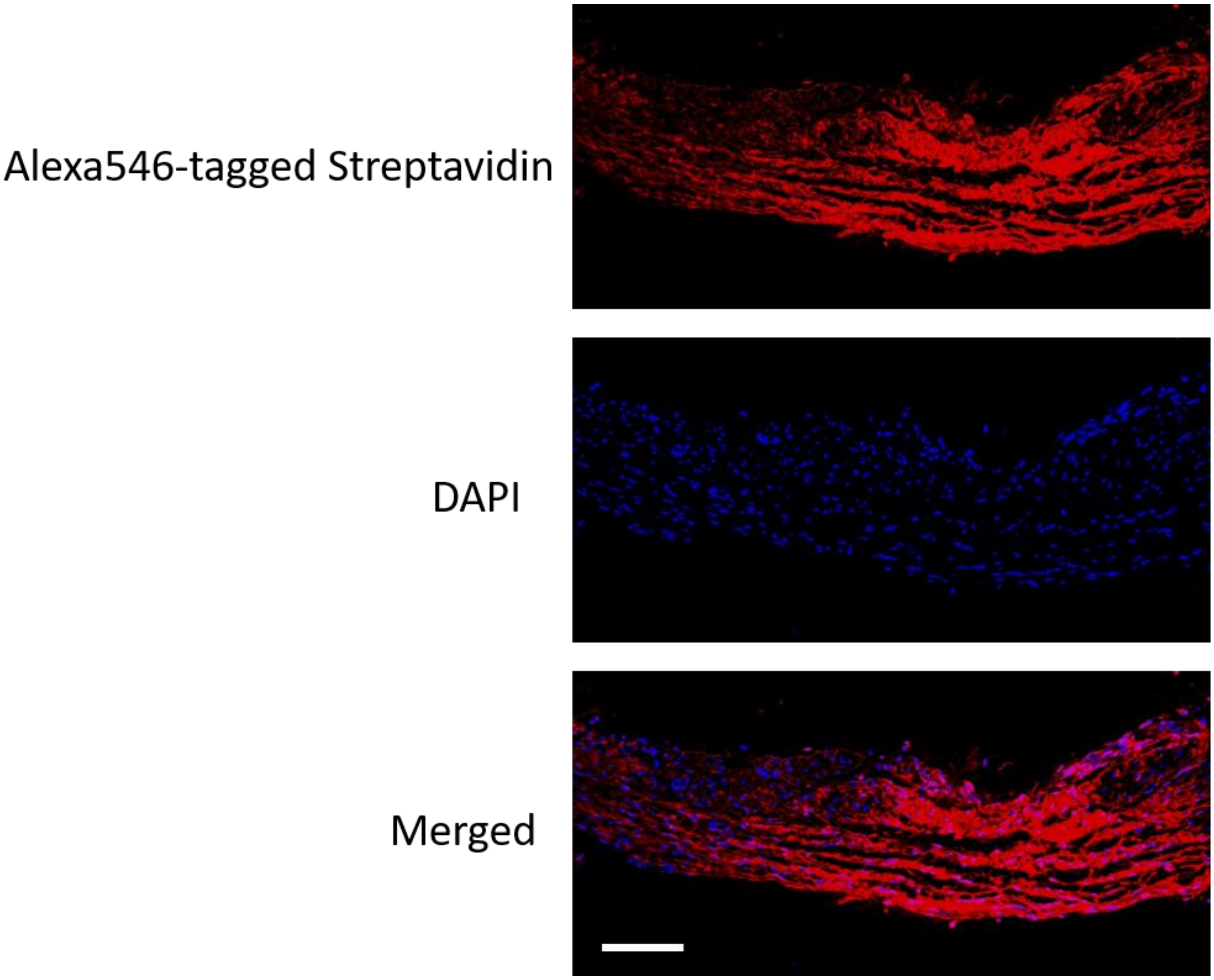

Suppl. Fig. 2. *Validation of the UBSM surface biotinylation protocol.* Rat UBSM was biotinylated, formalin-fixed, and paraffin-embedded. Sections were then processed for immunofluorescence imaging using Alexa546-tagged Streptavidin (red) and DAPI (blue) which show that the biotin-tagged reagents penetrate all layers of UBSM. Scale bar = 100 µm.

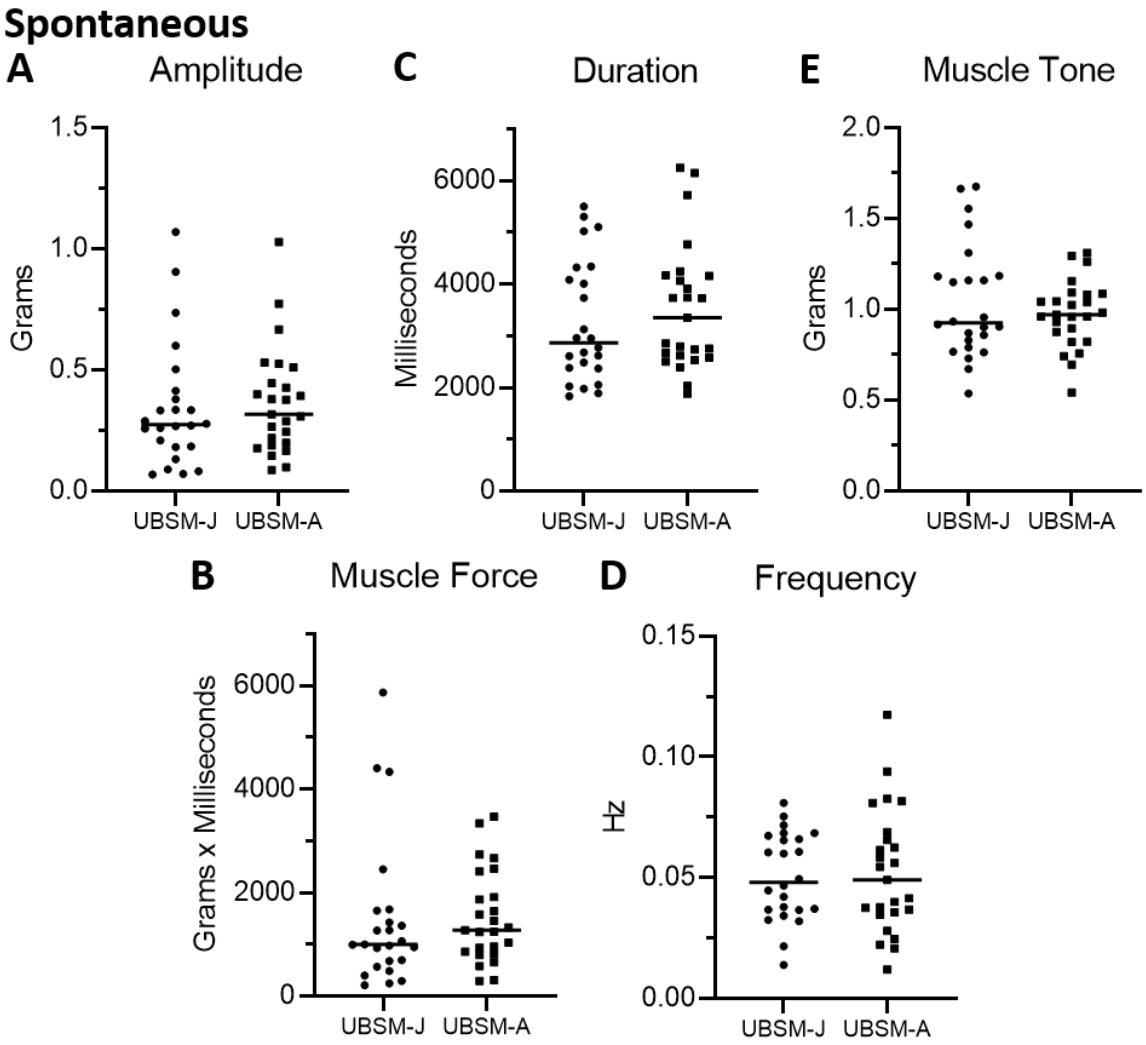

Suppl. Fig. 3. *UBSM-J and UBSM-A showed no discernible difference in the development of spontaneous phasic contractions* **A-E)** Illustrated are the control values of each contraction parameter for spontaneous phasic contractions used (Spontaneous: UBSM-J: *n*=24, *N*=11; UBSM-A: *n*=25, *N*=16). Values have not been normalized based on weight of individual tissue strips. Bars shown for each group indicate the median value for the indicated data points.

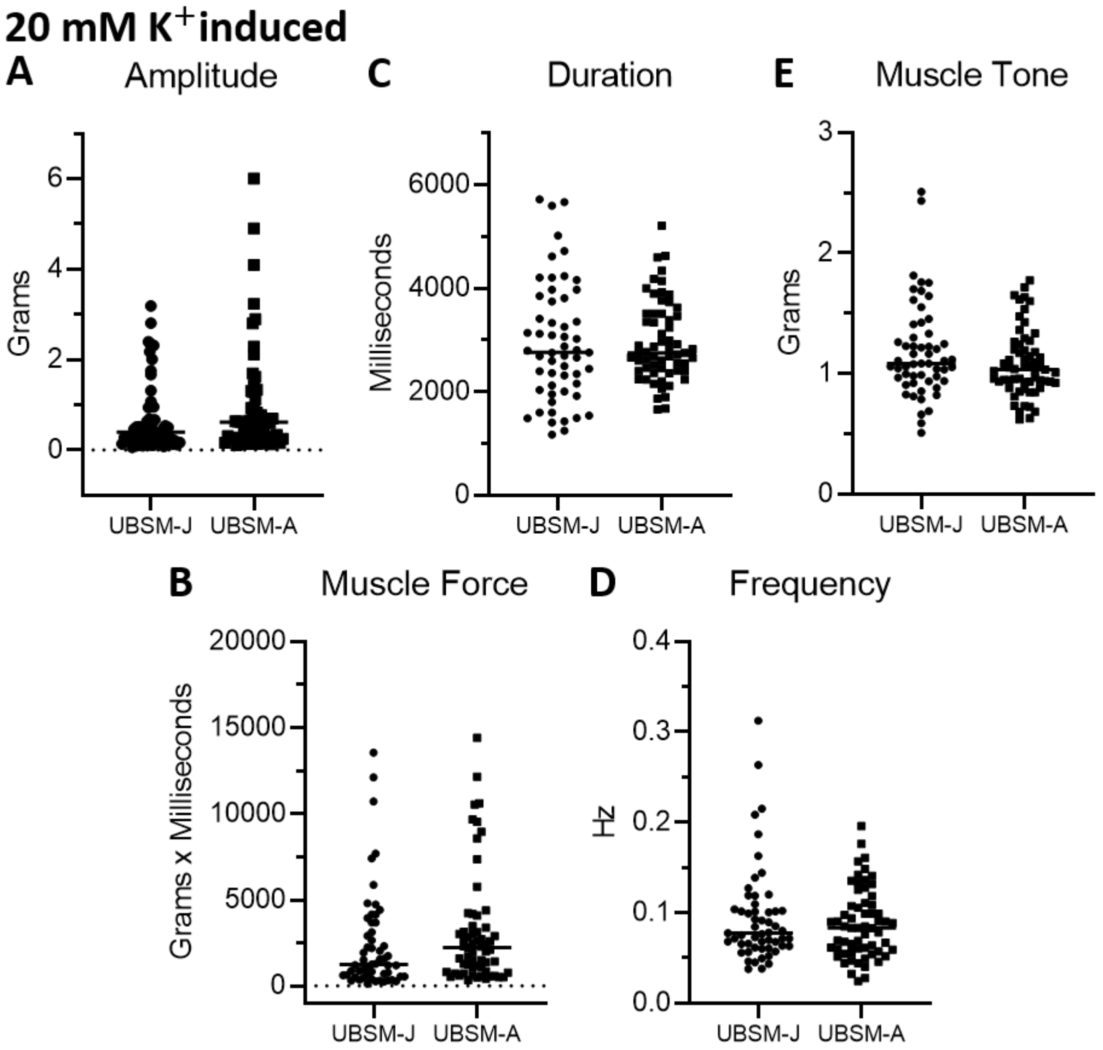

Suppl. Fig. 4. *UBSM-J and UBSM-A showed no discernible difference in the development of 20 mM KCl-induced contraction response.* **A-E)** Illustrated are the control values for each contraction parameter for 20 mM KCl-induced contractions (20 mM KCl-induced: UBSM-J: *n*=55, *N*=21; UBSM-A: *n*=61, *N*=21). Values have not been normalized based on weight of individual tissue strips. Bars shown for each group indicate the median value for the indicated data points.
